# A comprehensive sexual health care program for educable intellectually disabled adolescent girls: protocol for a mixed methods study

**DOI:** 10.1186/s12978-018-0587-3

**Published:** 2018-08-22

**Authors:** Shadi Goli, Mahnaz Noroozi, Mehrdad Salehi

**Affiliations:** 1 Student Research Center, Faculty of Nursing and Midwifery, Isfahan University of Medical Sciences, Isfahan, Iran; 2 Department of Midwifery and Reproductive Health, School of Nursing and Midwifery, Isfahan University of Medical Sciences, Isfahan, Iran; 3 Medical School, Isfahan University of Medical Sciences, Isfahan, Iran

**Keywords:** Educable intellectually disabled, Sexual health, Sexual problems, Needs, Mixed methods study, Intervention program, Health promotion

## Abstract

**Background:**

Protection and promotion of sexual health is of great importance for educable intellectually disabled adolescent girls; since they are prone to high risk sexual vulnerabilities and consequences, such as unwanted pregnancy, sexually transmitted infections, and acquired immune deficiency syndrome. Although the rights of intellectually disabled adolescents have been emphasized through the recent years, their sexual health care is still a challenge for parents, teachers, caregivers, and service providers. This study aims to present a comprehensive sexual health care program for educable intellectually disabled adolescent girls.

**Methods:**

This study is carried out by an exploratory sequential mixed qualitative-quantitative methods approach including three sequential phases. The researcher represents sexual health state of educable intellectually disabled adolescent girls using a qualitative approach. In the onset of the second phase, a comprehensive sexual health care program is designed for educable intellectually disabled adolescent girls. In this regard, in addition to qualitative studies, some related papers and texts are used. The suggested program of expert panel is approved based on prioritization guidelines. Then, in the third phase and after different stages of finalization of the program, its affectability is evaluated regarding improvement of sexual health state of educable intellectually disabled adolescent girls.

**Discussion:**

It is expected that from the results of the present mixed methods study, by presenting a comprehensive sexual health program for educable intellectually disabled adolescent girls, lead to improvements in the sexual health of these girls. Moreover, it wants to reduce risky sexual behaviors, sexual abuse and harassment, and their consequences in adolescent girls in order to improve sexual health state of the society. If this program works, it can become one of the leading education and care guidelines for sexual health care of intellectually disabled adolescent girls.

**Trial registration:**

IRCT20160224026756N5. Registered 22 June 2018.

## Plain English summary

Intellectually disabled adolescents, as well as normal people, experience different changes, such as secondary sexual characteristics and sexual desires. However, these changes may expose them to more problems and challenges due to their low intelligence quotient (IQ). Their poor understanding of inappropriate behaviors, inability in differentiating between malicious and normal sexual relations, problems in establishing negotiating relationships, and problem with reporting sexual abuses are some of the examples of their sexual vulnerabilities. These sexual harassments may be followed by unwanted pregnancies, sexually transmitted infections (STIs), human immunodeficiency virus and acquired immune deficiency syndrome (HIV/AIDS). Frequencies of these sexual abuses ultimately cause depression and anxiety in the adolescents. The findings of this study are suitable sources for selecting the best intervention of sexual health care of educable intellectually disabled adolescent girls. This study is carried out by an exploratory sequential mixed qualitative-quantitative methods approach including three sequential phases.

In this study, the researcher represents sexual health state of educable intellectually disabled adolescent girls using a qualitative approach. In the onset of the second phase, a comprehensive sexual health care program is designed for educable intellectually disabled adolescent girls. In addition to qualitative studies, some related papers and texts are studied. The suggested program of expert panel is approved and validated based on prioritization guidelines. In the third phase and after different stages of finalization of the program, its affectability is evaluated regarding improvement of sexual health state of educable intellectually disabled adolescent girls. It is expected that from the results of the present mixed methods study, by presenting a comprehensive sexual health program for educable intellectually disabled adolescent girls, lead to improvements in the sexual health of these girls. Moreover, it wants to reduce risky sexual behaviors, sexual abuse and harassment, and their consequences in adolescent girls in order to improve sexual health of the society.

## Background

Cultural and social beliefs limit the sexuality of intellectually disabled adolescents [1–3]. These intellectual problems cause them more physical disabilities than mental disabilities. Unfortunately, the public opinion about these child-like intellectually disabled adolescents is that they are bereft of any kind of sexual desires [4]. As a consequence, they are prevented from learning the necessary skills required for making informed choices about sexual issues. Therefore, they are exposed to abuse and rape risks [1, 5, 6]. Since these adolescents cannot differentiate between violent and non-violent relationships [7] and have the typical characteristics of passivity, obedience, and kindness, they are highly prone to sexual abuses and harassments [8, 9]. Sexual abuses can be followed by long term consequences, such as incompatibility, poor sexual performance, unwanted pregnancies, STIs, and HIV/AIDS [10–13]. Frequency of the sexual abuses ultimately causes depression and anxiety in the adolescents [11].

Intellectual disability is refereed to lower than average public intelligence (IQ < 70) which results in simultaneous destruction of adoptive behavior during the developmental phase and before the age of 18 [14]. Educable intellectually disabled people (their IQ ranges from 50 to 55 to 70) are able to continue their education until the sixth grade of elementary school and can obtain an acceptable level of independency and social adjustment by a limited support. Community-based exposure estimates for intellectual disability range from 10 to 15 per thousand children [15].

It should be noted that intellectually disabled adolescents, as well as normal people, experience different changes which occur in this age, such as secondary sexual characteristics and sexual desires [16–20]. However, these changes may expose them to more problems and challenges due to their low IQ [21, 22]. According to study findings, a wide range of sexual problems and manifestations can be observed in intellectually disabled adolescents, such as voyeurism, masturbation, divulging sexual desires in the public, expressing a desire to have sexual relationships with others and even with close ones, sexual abuse, poor understanding of inappropriate behaviors, inability in differentiating between malicious and normal sexual relations, problems in establishing negotiating relationships, and problem with reporting sexual abuses [22–25]. According to the researches, social and cultural context is an obstacle for discovering sexual desires of intellectually disabled adolescents. Although the rights of intellectually disabled adolescents have been emphasized through the last 40 years, their sexual health care is still a challenge for parents, teachers, caregivers, and service providers [26–28].

Although a lot of studies have been done on sexual problems of intellectually disabled adolescents and children in different countries around the world, there is a scarcity of them in Iran, especially studies using quantitative methodology and questionnaires. Since quantitative approach is limited in explaining some of the phenomena, such as humanistic values, cultural values, and human relations, it should be mixed with a qualitative approach in order to for the researcher to be able to understand deep and internal realities of human beings [29]. Therefore, with regard to different social, cultural, and religious contexts of Iran in comparison with other countries, a mixed (qualitative-quantitative) methods approach is used for identifying sexual needs and problems of educable intellectually disabled adolescent girls in order to design and execute a comprehensive sexual health care program for them.

### Objectives

Objectives of each phase are as the following:

#### Objectives of the first phase: qualitative study

Explaining sexual health status of educable intellectually disabled adolescent girls.

#### Objectives of the second phase: program design

Designing a primary intervention program based on extracted data from qualitative phase and reviews.

#### Objectives of the third phase: quantitative study

Explaining affectability of the comprehensive sexual health care program for educable intellectually disabled adolescent girls.

## Methods/Design

**Fig. 1:**
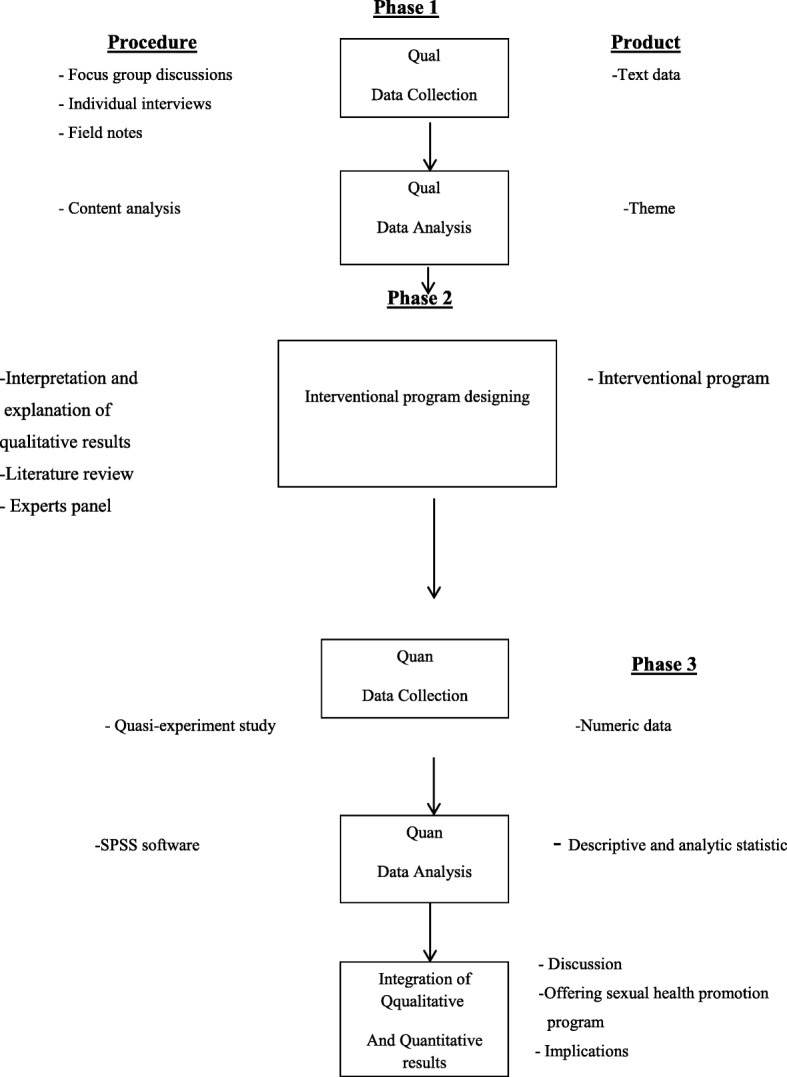
Study visual diagram

This study is carried out using an exploratory sequential mixed qualitative-quantitative methods approach including three sequential phases. The researchers use a qualitative approach to explain sexual health status of educable intellectually disabled adolescent girls and the required strategies for their sexual health care. For this end, they analyze understandings and experiences of the participants. In this phase of the qualitative study, the data are collected via semi-structured individual in-depth interviews; focus group discussions, and field noting. The participants are selected with a purposeful approach and with maximum variety. Simultaneous with data collection, the interviews will be analyzed with a conventional qualitative content analysis method. Sampling will be continued until saturation occured. After saturation and at the end of interviews, the researcher enters the second phase of the study in which a comprehensive program will be designed for sexual health care of educable intellectually disabled adolescent girls. For this end, a literature review of papers and texts will be used in addition to the results of qualitative study. Then, the suggested program will be validated based on prioritization guidelines. In the third phase and after different stages of finalization of the program, its affectability is evaluated regarding improvement of sexual health state of educable intellectually disabled adolescent girls. The collected data will be processed by SPSS Version 17.0 software and will be analyzed with descriptive-analytic statistical methods (Fig. 1).

### First phase: qualitative study

The first phase of this study is designed for answering the question of: “How is the sexual health status of educable intellectually disabled adolescent girls from the viewpoint of participants?”

This study will be carried out using a qualitative content analysis method.

#### Participants in the qualitative phase

Research community of the first phase consists of educable intellectually disabled adolescent girls, parents, teachers, mentors, psychologists, psychiatrists, consultants, gynecologists, midwives, and forensic medicine specialists. All of them have the experience of dealing with sexual problems of educable intellectually disabled adolescent girls.

#### Sampling method

In the present study, participants are selected with a purposeful sampling method and with maximum variety. They are selected based on maximum variety in age, education, social status, and job.

#### Inclusion criteria for participants

1. Tendency to participate in the study with informed consent for sharing information and participating in communication and interviews.
2. Iranian citizenship and the ability to understand and speak in Farsi.
3. No history of well-known psychiatric disorder.

#### Research environment

The participants are accessed through rehabilitation centers covered by the Ministry of Health and Welfare, exceptional education schools, consulting centers, hospitals, clinics, forensic medical centers, and psychiatrists’, gynecologists’, midwives’, and psychologists’ offices. The interviews will be done in the location and time selected by the participants for their ease and comfort.

#### Data collection process

After obtaining permission from Isfahan University of Medical Sciences, the researchers will select the participants by referring to research environments. In the qualitative phase, data collection methods include individual, in-depth, open, and semi-structured interviews along with focus group discussions and field noting. The interviews will be recorded. After explaining objectives and methodology of the study, the researcher will receive written consent regarding participation in the research, further interviews, and recording the interviews. Location of the interviews will be selected by the participants. If a person does not agree with recording, the researcher should try to create a friendly relationship and convince the person to consent to the recording procedure. In the next step, the cause of concern will be examined. If the participant’s concern is due to confidentiality, the researcher will try to convince him/her that confidentiality is fully respected. Finally, if the participant insists on this decision, the interview should not be recorded, but noting is allowed.

In semi-structured individual interviews, the first interviews are done with the aim of understanding possible and unpredictable issues. The general questions of semi-structured interviews are identified based on the resulted information. At the end of each interview, the narrative will be transcribed immediately, and data analysis will be done simultaneously with data collection. Data collection continues until saturation - as long as no new code or data is extracted. At this point, data saturation and adequacy will be verified.

#### Data analysis

A conventional content analysis method, introduced by Graneheim & Lundman (2004), is used for data analysis. Each interview will be transcribed immediately at the end of recording. After extracting the general idea, the narrative will be analyzed line by line, and its meaning units will be identified. Compact meaning units and codes will be extracted from these meaning units. After extracting the primary code, the data will be reduced and divided into sub- categories and main categories, based on their appearance [30].

#### Rigor and trustworthiness

For reliability and validity analysis, four criteria are suggested: credibility, dependability, transferability, and confirmability [31]. Different measures will be taken into consideration in order to improve credibility of this study, such as selecting the participants with maximum variety, spending sufficient amount of time on data collection, performing in-depth interviews in different locations and times, and mixing multiple data collection methods including individual interviews, focus group discussions, and field noting. For verification of extracted codes and data or their modification, they will be reviewed by the participants.

For confirming the reliability of the findings, some examples of code extraction methods and their corresponding interview narratives will be reviewed by an external supervisor in order to control the accuracy of researcher’s perception and to find contradictory cases. For increasing transferability, study findings will be presented to people who have similar characteristics with the participants in order to compare the results of this study with their own experiences.

Regarding verification, the researcher will explain the whole procedure, including recording, transcription, code extraction, and categorization. In order to verify the coding procedure, some of the research colleagues and faculty members, who are acquainted with qualitative research analysis and do not want to participate in this research, are asked to review the procedure.

### Second phase: designing the intervention program

After collecting the required data by qualitative study, the second phase of the study starts. The purpose of this phase is designing an intervention program for sexual health care of educable intellectually disabled adolescent girls. In this phase, the required strategies are extracted based on the results of qualitative phase and a literature review of papers and texts. These strategies will be validated by expert panel. Narrative review method is used for analyzing interview narrative which include electronic search in the area of the study. Moreover, the researcher will have access to library resources (reference books and dissertations) to obtain further information. Multiple databases are available for searching the related papers, such as Scopus, MEDLINE, Ovid, ProQuest, Cochrane Library, Science Direct, Web of Science, PubMed, Embase, Magiran, SID, Google Scholar, and CINAHL.

In this phase, all the English and Persian qualitative, quantitative, and mixed methods studies on educable intellectually disabled adolescent girls, sexual problems, sexual health of educable intellectually disabled adolescent girls, sexual health promotion, and adolescent sexual health care interventions, which have been published during 2006 to 2018, will be studied and analyzed. In the next phases, search will be narrowed down to specific mixed keywords, including educable intellectually disabled and “sexual health”, educable intellectually disabled and “sexual problems”, educable intellectually disabled and “sexual health needs”, educable intellectually disabled and “changes in puberty”, educable intellectually disabled and “sexual experience”, educable intellectually disabled and “sexual understanding”, educable intellectually disabled and “sexual health promotion”, educable intellectually disabled and “elimination of sexual problems”, educable intellectually disabled and “risk management”.

#### Holding a panel of experts

The objectives of this phase is to extract relevant strategies of sexual health care of adolescent girls from qualitative study and analyze the interview narratives in expert panel in order to prioritize their function. Then, the suitable intervention program will be selected and executed in the quantitative phase based on this prioritization. For this end, a decision making matrix will be extracted for prioritization of the extracted strategies from qualitative study and literature review of papers and texts. In this matrix, each strategy is provided with a score from 1 to 3 based on three criteria of costs, ease of execution, and time. This matrix will be sent for some specialists in the Delphi Round 1, average score will be calculated for each solution, high priority strategies will be identified, and consequently, a suitable intervention method will be selected. In Delphi Round 2, the selected intervention method will be evaluated qualitatively in a meeting with the presence of research team and panel members (psychiatric specialists, psychologists, gynecologists, reproductive health professionals, midwives, teachers, mentors, and authorities of The Ministries of Health and Welfare and The Ministries of Education). A copy of the intervention program will be presented to the experts who will be returned to the researcher at the end of the meeting. Comments and suggestions will be collected and applied in order to finalize and execute the program in the third phase (quantitative study).

### Third phase: quantitative study

#### Type of quantitative study

Quantitative phase of the study will be carried out using a three-group clinical trial.

#### Research population

The targeted populations for quantitative study are all the mothers of educable intellectually disabled adolescent girls who have referred to rehabilitation centers covered by The Ministry of Health and Welfare, and professional and pre-professional secondary schools covered by Special Education Organization of Isfahan.

#### Research sample

Study sample includes mothers of educable intellectually disabled adolescent girls who will be selected by convenience sampling and have all the inclusion criteria.

#### Research environment

This study will be carried out in three Girl’s Rehabilitation Centers covered by The Ministry of Health and Welfare, two professional secondary schools, and one pre-professional secondary school covered by The Ministry of Education in Isfahan. The reason for selecting such kind of environment is easy access to mothers of educable intellectually disabled adolescent girls and characteristics of the research unit.

#### Sample size

Sample size will be 27 participants in each group considering 95% confidence interval, 80% trial power, d = 2.9 for awareness, d = 11.3 for attitude, d = 5 for mothers’ self-efficacy, and 10% loss.

#### Sampling method

This clinical trial has two intervention groups and one control group. After identifying the centers covered by The Ministry of Health and Welfare and professional and pre-professional secondary exceptional schools, three rehabilitation centers covered by The Ministry of Health and Welfare and three exceptional secondary schools (two professional secondary schools and one pre-professional secondary school) will be selected non-randomly. Among these six centers and based on ALLOCATION and RANDOM software, one exceptional school and one rehabilitation center covered by The Ministry of Health and Welfare will be selected for each of the intervention group 1, intervention group 2, and the control group. Afterwards, the mothers with inclusion criteria will be selected from each center via convenience sampling method.

#### Inclusion criteria

1. Parents’ education should not be in the fields of medical sciences and psychology.
2. Mothers who have not previously been trained in adolescent sexual health education, child sexual abuse prevention, or sexual education courses.
3. Mothers should be able to read and write.

#### Exclusion criteria

1. Unwillingness to continue cooperation during the trial
2. Failure to receive 50% of the intervention for any reason

#### Research variables

In this clinical trial, the designed interventions are considered to be independent variables, and awareness, attitude, and mothers’ self-efficacy in the field of sexual health care of educable intellectually disabled adolescent girls are considered to be dependent variables.

#### Data collection methods

Questionnaires evaluating awareness and attitude, and mothers’ self-efficacy in the field of sexual health care of educable intellectually disabled adolescent girls are used in the quantitative phase of this trial. Researcher-made questionnaires are used for evaluation of mothers’ awareness and attitude. Reliability and validity of these questionnaires will be determined. Moreover, general self-efficacy questionnaire, introduced by Schwarzer & Jerusalem, is used for evaluation of mothers’ self-efficacy [32]. Mothers’ awareness questionnaire includes 23 questions with three options of “true, false, I do not know”. In measuring awareness score, one score will be given to the participants for each true answer. Minimum and maximum awareness scores are 0 and 23, respectively. Mothers’ attitude questionnaire includes 18 questions with a 5-point Likert scale of “strongly disagree, disagree, neither agree nor disagree, agree, and strongly agree”. Scoring is done from 0 to 4. Minimum and maximum attitude scores are 0 and 72, respectively. Self-efficacy questionnaire includes 10 questions with 4 options of “never true, rarely true, sometimes but infrequently true, and always true”. In measuring self-efficacy score, one score will be given to the participants for each true answer. Minimum and maximum self-efficacy scores are 0 and 10, respectively.

#### The implementation method

The researcher will implement the designed program after obtaining permission from ethics committee of Isfahan University of Medical Sciences and the necessary coordination with authorities of The Ministry of Health and Welfare and The Ministry of Exceptional Education. This is done by referring to the centers covered by The Ministry of Health and Welfare and secondary exceptional schools and presenting a letter of introduction to the authorities of those centers regarding research objectives. After that, the researcher will be allowed to access to phone numbers of girls in secondary educational schools and centers covered by The Ministry of Health and Welfare, and call their mothers in order to explain research objectives and invite them to participate in the research. If the mothers are willing to cooperate, and have all the inclusion criteria, they will be selected by convenience sampling method with their informed consent. Mothers’ awareness, attitude, and self-efficacy will be considered as the suggested outcomes. Before intervention, the questionnaires will be filled by mothers in each of the three groups. Furthermore, the questionnaires will be refilled by mothers immediately and 1 month after the intervention.

#### Data analysis

The collected data will be analyzed by descriptive statistical methods (mean, standard deviation, minimum, and maximum), inferential statistics (paired t-test, Chi-squared test, Fishers exact test, ANOVA, Wilcoxon test, and Mann-Whitney test), and SPSS v20 software. ANCOVA will be used for adjusting intervening variables.

#### Integration of the qualitative and quantitative data

The results of the qualitative and quantitative phases of the study will be integrated and finally, a program for sexual health of educable intellectually disabled adolescent girls will be provided.

## Discussion

Multiple researches indicate that intellectually disabled adolescents need a comprehensive sexual health care program in order to be protected from sexual abuse and harassment and their adverse consequences, such as unwanted pregnancy, illegal abortions, STIs, and HIV/AIDS [33–39]. Negative attitudes of teachers and mentors toward sexual education of intellectually disabled adolescents, cultural obstacles, parent’s awareness and attitude, and sexual taboos have limited the design and implementation of such kind of comprehensive programs and have caused inattention to sexual health care of intellectually disabled adolescents [22, 40–42].

Moreover, the researchers have supported studies carried out on education interventions of parents of intellectually disabled adolescents as a sexual health care strategy which will reduce sexual abuse and harassment consequences [22, 43]. According to the importance of sexual health care of intellectually disabled adolescents, the present study aims to design and implement a comprehensive sexual health care program for these adolescents. The framework of this program will improve awareness, attitude, and self-efficacy of mothers in the field of sexual health care. So they can help their girls to have healthy relationships, protect themselves in unhealthy situations, and prepare themselves for a healthy, safe, and socially accepted sexual life. This mixed methods study and its resulted intervention program should be able to improve sexual status of these adolescent girls and reduce the consequences of sexual abuse and harassment, malicious sexual behaviors in them, and consequently, improve social health. If this program works, it can become one of the leading education and care guidelines for sexual health care of intellectually disabled adolescent girls.

## Abbreviations

AIDS: Acquired immune deficiency syndrome
HIV: Human immunodeficiency virus
IQ: Intelligence quotient
STIs: Sexually transmitted infections
